# Familial Mediterranean Fever in Iran: A Report from FMF Registration Center

**DOI:** 10.1155/2015/912137

**Published:** 2015-08-27

**Authors:** Farhad Salehzadeh

**Affiliations:** Pediatric Department, Bouali Children's Hospital, Ardabil University of Medical Sciences (ARUMS), No. 105 Shahrak Azadi Azarbyjan Street, Ardabil 56157, Iran

## Abstract

*Background*. Familial Mediterranean fever (FMF) is a periodic AR autoinflammatory disorder. This comprehensive study describes FMF in Iran as a country near Mediterranean area. *Materials and Methods*. From the country FMF registration center 403 patients according to Tel-Hashomer criteria enrolled this study, 239 patients had MEFV gene mutations analyses. Data, if needed, was analyzed by SPSS v20. *Results*. 175 patients (43.4%) were female and 228 patients (56.6%) were male. The mean age was 21.3 years. Abdominal pain was in 93.3% patients and 88.1% had fever. Abdominal pain was the main complaint of patients in (49.6%). The mean interval between attacks was 36.5 ± 29.6 days and the mean duration of every episodes was 43.3 ± 34.5 hours. 15.1% of patients had positive family history and 12.7% had previous surgery; in 52.3% of patients delay in diagnosis was more than three years. 12 common MEFV gene mutations were analyzed, 21.33% were without mutations, 39.7% had compound heterozygote, 25.52% showed heterozygous, and 13.38% showed homozygous results. The most common compound genotype was M694V-V726A (% 10.46) and in alleles M694V (% 20.9) and V726A (% 12.7) were the most frequent mutations, respectively. *Conclusion*. M694V was the most common mutation, and the most common compound genotype was M694V-V726A. Our genotype results are similar to Arabs and in some way to Armenians, erysipelas-like skin lesions are not common in this area, and clinical criteria are the preferred methods in diagnosis of FMF.

## 1. Introduction

Familial Mediterranean fever (FMF) is an autosomal recessive inherited disorder [1, 2]. It is common among Mediterranean populations (Jews, Arabs, Turks, and Armenians) [3]. FMF is characterized by recurrent fever and inflammation of serous membranes, leading to abdominal pain, joint pain, and chest pain.

The most important complication of FMF is amyloidosis, which eventually leads to kidney failure [4]. Symptoms of the disease usually occur during the first decade of life in more than 80% of patients [5]. The highest prevalence of the disease among the “Sephardic Jews” and the Armenians has been reported [6].

FMF seems to be more common in males with the prevalence rate 1.5 to 2 times [7]. The disease frequency at an early age and infants is low [8]. In Middle East its prevalence is one in 2,000 to one in 100, according to different studies [9].

MEFV gene, responsible for the disease, located on chromosome 16 P (13.3) codes synthesis of a protein called pyrin [10].

Ongoing and recurrent inflammation leads to amyloid production and deposition of it in vital organs, especially the kidneys [7].

More than 300 known mutations in the MEFV gene have been reported [11].

The appearance of homozygous mutations was significantly associated with severe clinical presentations of the FMF [10, 12]. This mutation is related to disease onset at an early age and more prevalence of arthritis and progression to amyloidosis [13].

M680I is common among Armenians and Turks and is associated with more severe form of the relapsing fever in early childhood which may be the only presentation of familial Mediterranean fever [2]. In 90% of the patients disease is begun before 20 years of age [9].

Approximately 50% of patients experience joint pain during the attacks. The pain is usually limited to one joint at a time [9]. Typical FMF arthritis is usually benign and self-limited acute monoarthritis [14]. The most commonly involved joints include hip, knees, elbows, and wrists [14–16].

Protracted febrile myalgia syndrome (PFMS) is presented with prolonged fever and an increase of ESR and leukocytosis. It usually takes 6 to 8 weeks and respond to treatment with prednisolone [17, 18]. The M694V mutation associated with a higher incidence of PFMS [19].

About 45% of patients have experienced pleural attacks. Pleural attacks are sudden, unilateral attacks that subsides within 48 hours [20].

Pleuritis prevalence has been between 20% and 80% in different studies [7]. Pericarditis and tamponade, myocarditis, and cardiac amyloidosis are the most common cardiovascular presentation in FMF [21, 22].

The nonamyloidosis renal involvement has been reported in 22% of patients and include temporary or permanent hematuria, proteinuria, acute pyelonephritis, interstitial nephritis, and glomerulonephritis due to coexisting disease [23, 24].

The classic skin lesion is periodic erysipelas-like skin rash. It is a pathognomonic finding in FMF which usually fades within 72–48 hours; fever and leukocytosis may accompany the rash. The incidence of rash is 3%–46% and is usually associated with M694V mutation [15]; sometimes erysipelas rash and fever are the only sign of FMF [25].

Acute scrotal swelling, a painful inflammation of the testis, in a small percentage of children with FMF, has been reported. In Arabs it has been described as a common feature of FMF [26]. Infertility in patients with FMF has no difference with the general population [27, 28]. Recurrent aseptic meningitis can rarely occur in FMF; these attacks are prevented after treatment with colchicine [29].

Familial Mediterranean fever may be associated with different systemic inflammatory diseases including Behcet, polyarteritis nodosa and Henoch-Schonlein purpura [30], spondyloarthropathy [2], multiple sclerosis [31, 32], and inflammatory bowel diseases [33, 34].

On the basis of clinical findings Tel-Hashomer criteria have been described to be FMF diagnosis [11, 35].

There is no specific standard laboratory test for FMF; nowadays compound MEFV gene mutations in hemo- or heterozygote forms confirm the diagnosis of FMF [36].

## 2. Materials and Methods

This is a case series study. This study included all patients with familial Mediterranean fever in FMF Clinic at Bouali Hospital and FMF Registration Center in Iran (http://www.fmfiran.ir/). 422 patients were enrolled in this study; all patients had FMF according to Tel-Hashomer criteria and/or at least two MEFV gene mutations. 19 patients were excluded because of unavailability and study was conducted on 403 patients with FMF. Common MEFV genes mutations (E148Q, P369S, F479L, M680I (G/C), M680I (G/A), I692del, M694V, M694I, K695R, V726A, A744S, and R761H) have been analyzed in 239 patient. Descriptive analysis was used to analyze data by V20 SPSS.

## 3. Results

The mean age of patients was 21.03 years. The youngest patient was 1.5 years old and the oldest one was 76 years old. The average age of onset of symptoms was 9.75 years and the first decade of life was the most common age range which was in 128 patients (31.8%).

228 patients (56.6%) were males and 333 patients were living in urban area. The main complaint of patients was abdominal pain, in 200 cases (49.6%), and abdominal pain plus fever in 67 (16.6%) patients was the second main complaint.  Table 1 shows the main symptoms of patients.

Interval between attacks was 36.5 ± 29.6 days and the mean duration of each episode was 43.3 ± 34.5 hours.

239 patients had MEFV gene mutations analysis; Table 2 shows these results. 51 patients (21.33%) had no mutations in this analysis.

The most common homozygous mutation was M694V-M694V (7.1%) and compound heterozygous mutation was M694V-V726A (10.46%) and monoheterozygous mutation was E148Q (8.7%).

Genotype-phenotype correlation has been shown in Table 3.

With regard to the time between onset of symptoms and diagnosis of FMF in 207 patients (52.3%) this period was more than 3 years.

Colchicine administration in 89.9% patients had good results and in 7.2% patients showed moderate response while 2.9% patients had poor response. 13.2% of patients used drug irregularly. The most common colchicine side effect was diarrhea in 4.7% of patients.

In our series two cases had erysipelas-like skin rash with negative MEFV results which indicate the rarity of this feature in this area. We had two siblings with idiopathic hepatitis and massive ascites who were homozygous for M694V gene.

## 4. Discussion

Many studies have shown male preponderance in FMF [38, 40, 41]. In Sackesen et al. [52] and Dunder et al. [37] the average age of onset was second decade of life. In Yilmaz study [38] 90% of patients had fever, 95% of patients had abdominal pain, and 19% of patients had erroneous appendectomy. In a study on 229 patients, 115 were females and 68% of patients had MEFV gene mutations. High fever in 94% of patients and abdominal pain in 83% were the main symptoms [53].

In Ebrahimi-Fakhari et al. [54] fever in 78% of patients, peritonitis symptoms in 95%, pleuritis in 59%, arthritis in 32%, arthralgia in 60%, erysipelas-like skin rash in 23%, vasculitis in 8% have been reported.

In Dundar's [37] report 49.6% of patients had no MEFV mutations, 26.41% were heterozygous, 15.29% were compound, and 8.60% were homozygous mutation. In Balci et al. [55], Esmaeili et al. [41], and Bonyadi et al. [40] case series results have been shown in Table 4. Comparison of this study with different ethnic groups has been presented in Table 5.

The genetic expression of this study is similar to Arabs and somewhat to Armenians.

In this study, the common combined heterozygous mutation was M694V-V726A with 10.46%, M694V-M694V with 7.1%, and M680I-V726A with 6.27%. The most common genotype of patients was M694V-M694V in different studies [38, 40, 41]; this genotype was in second rate of our results.

In this study M694V mutation with 18.3% and compound mutation V726A-M694V with 12.7% were common mutations in male, while M694V mutation with 24.5% and M694V-M694V with 10.7% were the common genotype in female. The sexual genotype difference has not been mentioned in certain studies.

Rare mutations were observed in this study such as K695R and F479L with 0.2% frequency. In Manna et al. report [53] rare mutations were K695R with 0.67%, M694I with 1.04%, and R761H with 1.91%. In Yilmaz study [38] the R761S in 1% and V726A in 3% of patients were rare mutations.

The Bonyadi study [56] showed these rare mutations: A744S in 0.5%, M694I in 2.2%, F479L in 1.3%, P369S in 1.3%, E167D in 1.2%, and R408Q in 1.2%.

In our study there was one case of amyloidosis who presented with resistant nephrotic syndrome and M694I-M694I mutations.

Some studies have shown that the M694V mutation is associated with a severe form of the disease [55]. Kincir et al. showed that arthritis and abdominal pain accompany mutations M694V and E148Q [57].

This study showed that M694V-M694V mutations are more common in patients with presentation during first decade of life, while in those with disease presentation during second decade or later genotype V726A-M694V was common. Patients with homozygous mutation M694V have lower age at onset [16].

In our study two patients presented with the complaint of recurrent orchitis and compound heterozygous mutations M694V-V726A and M694V-E148Q, and two female siblings had massive ascites, abdominal pain, and idiopathic hepatitis with homozygous mutation M697V; this association has been reported in the literature [2].

## 5. Conclusion

This study showed that first decade of life is the most common age in disease presentation of FMF; abdominal pain is the main symptom; M694V and M694V-V726A are the most common alleles and compound genotype. M694V-M694V genotype is associated with lower age presentation and joint involvement. Our genotype is similar to Arabs and in some way Armenians and erysipelas-like skin rash is not common feature in our patients.

## Figures and Tables

**Table 1:** Clinical findings in our patients.

Clinical symptoms	Number of patients	Percent
Abdominal pain	376	93.3
Fever	355	88.1
Fatigue	282	70
Anorexia	258	64
Nausea	139	34.5
Myalgia	118	29.3
Vomiting	107	26.6
Arthralgia	103	25.6
Constipation	96	23.8
Chest Pain	72	17.9
Diarrhea	69	17.1
Vertigo	42	10.4
Dysuria	41	10.2
Headache	36	8.9
Arthritis	31	7.7

**Table 2:** Results of MEFV genes mutations and different allele's analysis.

Mutation	Genotype	Percent	Number
Heterozygote *N* = 61 (25.52%)	E148Q	8.7	**21**
	M694V	8.36	**20**
	V726A	2.5	**6 **
	A744S	1.7	**4 **
	M680I	2.1	**5**
	P396S	1.2	**3 **
	R761H	0.84	**2**

Homozygote *N* = 32 (13.38%)	M694V-M694V	7.1	**17**
	M680I-M680I	2.92	**7**
	E148Q-E148Q	0.84	**2 **
	M694I-M694I	0.84	**2**
	V726A-V726A	0.84	**2**
	P396S-P396S	0.42	**1**
	R761H-R761H	0.42	**1**

Compound heterozygote *N* = 95 (39.7%)	M694V-V726A	10.46	**25**
	E148Q-M694V	2.96	**7**
	M680I-V726A	6.27	**15**
	R761H-M694V	2.5	**6**
	M680I-M694V	2.5	**6**
	R761H-E148Q	0.42	**1 **
	V726A-R761H	2.5	**6**
	M694V-M694I	0.84	**2**
	M680I-R761H	2.5	**6 **
	M680I-E148Q	0.84	**2**
	E148Q-P396S	2.96	**7 **
	A744S-R761H	0.42	**1 **
	E148Q-V726A	1.69	**4**
	K694R-V726A	0.42	**1**
	E148Q-A744S	0.42	**1**
	E148Q-M694I	1.27	**3**
	M694I-M680I	0.42	**1**
	F497L-E148Q	0.42	**1**

Patient with no identified mutation *N* = 51 (21.33%)		21.33	**50**

**Table 3:** Genotype and phenotype correlations.

	Common combined mutations	The most common alleles
Abdominal pain *N* = 120	V726A-M694V *N* = 159 (12.5%)	M694V *N* = 47 (19.6%)

Fever *N* = 36	V726A-M680I *N* = 5 (13.9%)	M694V *N* = 14 (19.4%)

Chest pain *N* = 12	V726A-M694V and R761H-M694V *N* = 2 (16.7%)	M694V *N* = 7 (29.2%)

Joint pain *N* = 6	M694V-M694V *N* = 2 (33.3%)	M694V *N* = 6 (50%)

Patients with positive family history *N* = 38	V726A-M694V *N* = 10 (26.3%)	M694V *N* = 25 (32.9%)

Age at FMF onset 1 to 10 years *N* = 156	M694V-M694V *N* = 11 (7.1%)	M694V *N* = 58 (18.1%)

Age at FMF onset 10 to 20 years *N* = 41	V726A-M694V *N* = 5 (12.2%)	M694V *N* = 19 (23.2%)

Age at FMF onset after 20 years *N* = 42	V726A-M694V *N* = 10 (23.8%)	M694V *N* = 25 (29.8%)

Male *N* = 134	V726A-M694V *N* = 17 (12.7%)	M694V *N* = 49 (18.3%)

Female *N* = 106	M694V-M694V *N* = 11 (10.4%)	M694V *N* = 52 (24.5%)

**Table 4:** Comparison of different genotypes studies.

Studies	Mutations frequencies (%)				
	M694V	V726A	M680I	M694I	E148Q
Dundar et al. [37]	68.14	76.4	62.7	0.46	15.5
Yilmaz et al. [38]	55	3	16	0	10
Ben-Chetrit et al. [39]	38	4	8	0	4
Bonyadi et al. [40]	42.4	17	15.2	2.2	16.2
Esmaeili et al. [41]	28	9	7	1	7
Our study	20.9	12.7	10.3	2.1	10.7

**Table 5:** Comparison of different ethnics' studies.

Ethnic groups	M694V	V726A	M680I	M694I	E148Q
Jews [39, 42, 43]	77	12.3	0.6	0	10.2
Armenians [44, 45]	52	26	20	0.2	1.8
Arabs [46–48]	42.5	23.1	9.6	14.1	10.7
Turks [49–51]	71.3	8.5	15	1.7	3.5
Iranian, Azeri Turks [41]	54	16.7	12.6	2.5	14.2
Our study	20.9	12.7	10.3	2.1	10.7
